# Factors Influencing Frequency of Depressive Experiences Among Married Working Women in South Korea

**DOI:** 10.3390/healthcare13050453

**Published:** 2025-02-20

**Authors:** Se Hui Jeong, Chan Mi Kang, Kyung Im Kang

**Affiliations:** 1 Offshore Division, ELL, 101-201, Hwajam-ro 51, Dong-gu, Ulsan 52727, Republic of Korea; sehui.jeong@ellinterpretingenergy.com; 2 Department of Nursing, Division of Health Science, Dongseo University, 47 Jurye-ro, Sasang-gu, Busan 47011, Republic of Korea; chanmikang@gdsu.dongseo.ac.kr; 3 College of Nursing, Gyeongsang National University, 15 Jinju-daero 816, Jinju 52727, Republic of Korea

**Keywords:** depressive experiences, married working women, South Korea, zero-inflated negative binomial model

## Abstract

**Background/Objectives**: This study aimed to identify the factors influencing and predicting the frequency of depressive experiences among married working women in South Korea in the post-COVID-19 period (2022–2023). It examines how alterations in circumstances and the complex difficulties encountered by this demographic group may have shaped their depressive experiences. Through a comparative analysis of the group reporting depressive experiences and the group reporting no depressive experiences, the study delineates the factors influencing depressive experiences within the former group and the predictive factors within the latter group. The findings offer a comprehensive understanding of the factors that may contribute to mental health outcomes within this population. **Methods**: This study utilized data from the ninth wave (2022–2023) of the Korean Longitudinal Survey of Women and Families, conducted by the Korean Women’s Development Institute. The study included a total of 1735 participants. A zero-inflated negative binomial regression model was applied to analyze the frequency of depressive experiences and the influencing and predictive factors. **Results**: Among the participants, 38.9% reported no depressive experiences. The count model analysis revealed that subjective health status, physical activity, thoughts about husband, family decision-making, and work–family balance were significant factors associated with the frequency of depressive experiences. In the logistic model, key predictors for those without depression included the spouse’s education, physical activity, satisfaction with the spouse’s housework, and happiness with marital life. **Conclusions**: These findings provide essential empirical evidence for the development of targeted policies and interventions aimed at mitigating and preventing depression problem among married working women.

## 1. Introduction

The 2023 National Health Statistics-9th Year, 2nd Annual Report by the Korea Disease Control and Prevention Agency revealed that the rate of depressive experiences, a crucial mental health indicator, reflects the subjective degree to which individuals experience depressive symptoms such as sadness, hopelessness, or emotional distress that disrupts daily functioning. In 2023, the prevalence of depressive experiences was 14.7% among women and 8.7% among men, suggesting that women experience depression at a higher rate than their male counterparts. Upon further analysis of the longitudinal data, the average prevalence of depressive experiences for women was approximately 15.6%, compared to 8.9% for men, indicating that women experience depressive feelings approximately 1.8 times more frequently than men [1]. Notably, the COVID-19 pandemic has exacerbated this gender disparity in depressive experiences. A survey conducted by the Ministry of Health and Welfare, which included 2063 adults aged 19 to 71, revealed that the prevalence of depressive experiences among women was 18.6%, which is 3.3% higher than that of men (15.3%) [2].

In this context, as of March 2024, the economic participation rate of women in South Korea was 63%, reflecting an 8.1% increase over the past decade, according to the Monthly Economically Active Population Survey [3]. Despite this rise in women’s participation in economic activities, traditional gender roles within households remain virtually unchanged, with married working women continuing to bear the majority of responsibilities for housework, childcare, and elderly care [4]. Particularly during the COVID-19 pandemic, social distancing measures intensified gender role conflicts for married working women, resulting in heightened psychological stress and depressive experiences [5]. Depression among married working women may lead to long-term health issues, negatively impacting physical health and, eventually resulting in productivity loss due to frequent absenteeism or workforce withdrawal [6,7,8]. Therefore, in-depth research on the levels of depression experienced by married working women during and after the later phases of the COVID-19 pandemic, as well as the determining factors, is imperative.

Previous studies have classified the factors affecting depression among married working women into four main categories: (1) sociodemographic factors, including age, education, spouse’s education level, income level, and number of children [9,10,11]; (2) health perception and behavior factors, such as subjective health status, physical activity, BMI (Body Mass Index), and body shape perception [12,13,14]; (3) household perception and attitude factors, including satisfaction with the spouse’s contribution to housework, happiness with marital life, thoughts about husband, household decision-making, and family values [12,15,16]; and (4) workplace perception and attitude factors, such as employment type, job satisfaction, and work–family balance [9,11]. However, the findings of these studies, both domestic and international, have shown inconsistencies and a lack of coherence, making it difficult to draw definitive conclusions about the impact of these individual factors. Furthermore, limitations in data analysis were noted, owing to the statistical methods employed. In light of these challenges, applying more effective statistical methods that are better suited to the characteristics of the relevant data may provide a more precise understanding of the factors associated with depression among married working women.

Various Likert-type scales developed to measure the severity of depression often result in a high number of respondents reporting no depressive experiences, indicating that many respondents experience little to no depression, producing data that does not meet the normality assumption [17,18]. These data can be classified as count data, which include non-negative integer values starting from zero, representing the frequency of specific events, such as depression. When logistic regression analysis, commonly used in previous studies, is applied to this count data, several issues arise.

First, logistic regression is suitable for binary outcomes; however, count data may contain a range of integer values beyond zero, making it difficult to accurately model this distribution and potentially leading to information loss [19,20]. Second, an excessive number of zero values in a dataset might not simply indicate the absence of occurrences but could also suggest additional heterogeneity within the dataset. Logistic regression tends to fail to account for this characteristic of excess zeros, reducing prediction accuracy [21]. Third, in logistic regression, the coefficients of individual independent variables indicate their effect on the log odds of the outcome variable. However, for count data, this interpretation might not accurately represent the true structure of the dataset, potentially resulting in ambiguous interpretations or incorrect conclusions [22].

Therefore, as count data are better to analyze using a zero-inflated negative binomial (ZINB) regression model [23], depressive experiences, being considered as count data, warrant an attempt to apply the ZINB. The first model (count model) examines the factors associated with the occurrence of issues in the affected group (non-zero responses) [24]. This model identifies critical risk factors that influence both the frequency and intensity of depressive experiences among those who have reported such experiences. The second model (logit model) aims to identify predictive factors that mitigate the likelihood of depressive occurrences in the unaffected population (zero responses) [24]. This model targets individuals who have not reported any depressive experiences, aiming to elucidate significant predictors for potential future depressive experiences. This approach effectively analyzes a dataset with an excessive number of zero values, such as the frequency of depressive experiences, and helps identify predictors of both the severity and occurrence of depression simultaneously [25].

Consequently, this study applies the ZINB regression model using data from the ninth wave (2022–2023) of the Korean Longitudinal Survey of Women and Families to investigate the extent of depression experienced by married working women during and after the later phases of the COVID-19 pandemic, as well as to identify the associated factors. By investigating the groups reporting depressive experiences and those reporting no depressive experiences, this study seeks to identify the factors that influence depression within each group and to highlight the necessity for targeted interventions. Additionally, the study will identify specific predictive factors, thereby providing essential foundational data for the development of policies and programs tailored to the unique characteristics of each group. Furthermore, the findings will contribute to the formulation of comprehensive societal strategies aimed at preventing and supporting the mental health of married working women. Ultimately, this research will offer evidence-based recommendations to enhance the quality of life and improve productivity within this demographic, thereby fostering long-term positive societal outcomes.

## 2. Materials and Methods

### 2.1. Study Design

The current cross-sectional analysis is a secondary analysis of data from the ninth wave (2022–2023) of the Korean Longitudinal Survey of Women and Families, conducted by the Korean Women’s Development Institute.

### 2.2. Data Source and Participants

The data for this analysis were obtained from the ninth wave (2022–2023) of the Korean Longitudinal Survey of Women and Families, a nationwide panel survey conducted by the Korean Women’s Development Institute. This panel survey collected data in two rounds, the first in 2022 and the second in 2023, targeting women aged 19 years and older across South Korea. Sampling was based on a sampling frame of approximately 260,000 enumeration districts from the 2005 Population and Housing Census. The initial sampling units were stratified by region (municipalities and provinces), considering various variables such as urbanization level, employment rates by industry sector, housing type, household composition, and the age and sex of the household head. The probability proportional to the size sampling method was applied for participant selection.

**Figure 1:**
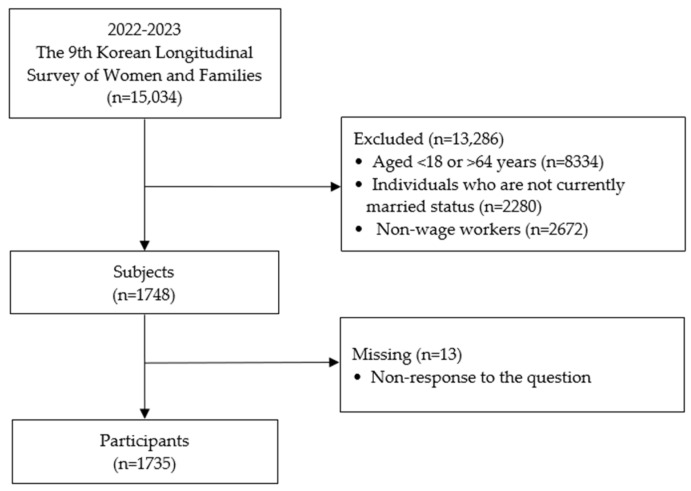
A flowchart of the sampling process in the study.

During the study period, an extra 5037 eligible household members were added to the initial sample of 9997 household members, resulting in a total of 15,034 participants. The inclusion criteria for this study were as follows: participants had to be currently married, aligning with the study’s focus on married women. Additionally, participants needed to be between 19 and 64 years of age, corresponding to the age range of married working women. Only wage workers were included, as the study specifically targeted employed individuals, excluding non-wage workers to focus on the demographic of married working women. Based on predefined criteria, 13,286 individuals were excluded: 8334 were outside the age range of 19 to 64 years, 2280 were not currently married, and 2672 were non-wage workers. Additionally, 13 cases were excluded due to incomplete responses to the question regarding their spouse’s education level. Following these exclusions, the final analytic sample consisted of 1735 participants, as depicted in Figure 1.

In this study, the inherent characteristics of public survey data were meticulously considered, with particular attention to the voluntary nature of respondent participation and the occurrence of missing data. During the analysis phase, it was concluded that imputing missing values could potentially introduce bias into the results. Specifically, in instances where respondents may have omitted answers due to sensitivity or reluctance to respond, the application of imputation techniques, such as mean imputation, could lead to distortion of the original data distribution. Consequently, rather than resorting to imputation, this study excluded missing values, which produced more consistent and reliable results. Given that public survey data does not permit the enforcement of responses, the removal of missing data is a widely accepted approach. This methodological decision was deemed necessary to ensure the integrity, accuracy, and consistency of the research findings.

### 2.3. Variables

#### 2.3.1. Outcome Variable: Frequency of Depressive Experiences

The frequency of depressive experiences over the past week was assessed using the CES-D 10 scale employed in the Korean Longitudinal Survey of Women and Families, which is a modified version of the Center for Epidemiological Studies Depression (CES-D) originally developed by Radloff [26]. The CES-D 10 scale consists of eight forward-scored items and two reverse-scored items (5 and 8): (1) I was bothered by things that usually don’t bother me, (2) I had trouble keeping my mind on what I was doing, (3) I felt depressed, (4) I felt that everything I did was an effort, (5) I felt hopeful about the future, (6) I felt fearful, (7) My sleep was restless, (8) I was happy, (9) I felt lonely, and (10) I could not get going. Each item was rated on a 4-point scale: 0 = rarely or never (less than 1 day); 1 = sometimes (1–2 days); 2 = moderately (3–4 days); and 3 = most or all of the time (5–7 days). The total score ranges from 0 to 30, with a higher score indicating a higher frequency of depressive experiences. A score of 10 or higher is commonly recognized as the threshold for clinically significant depression. However, instead of dichotomizing CES-D scores into a binary classification, this study distinguishes between zero responses, representing respondents who reported no depressive experiences, and non-zero responses, which utilize the full range of CES-D scores to assess both the frequency and severity of depressive experiences among those reporting such symptoms. This refined approach supports a more comprehensive analysis by facilitating the identification of factors influencing and predicting depressive experiences while maintaining the continuity of the data.

The reliability coefficient (Cronbach’s α) for the scale used in this study was calculated at 0.856.

#### 2.3.2. Explanatory Variables: General Characteristics of Participants

1. Sociodemographic factors include six variables: age, education, spouse’s education, monthly income, and the number of children (preschool and school-aged children). Age and monthly income were measured as continuous variables, while education level was categorized into three groups: middle school or lower, high school, and college or higher. The number of children was divided into two categories: preschool children and school-aged children (elementary to high school).
2. Health perception and behavior factors include three variables: subjective health status, physical activity, and body shape perception. Subjective health status was measured using a single item, through which respondents evaluated their own health in three categories: good, average, and poor. Physical activity was assessed with a single item measuring the frequency of intensive physical activity in the past week and was converted into a binary (yes/no) scale. BMI was derived from the collected height and weight data. Body shape perception was also assessed with a single item, through which participants evaluated their body shape in three categories: thin, average, and obese.
  Smoking and drinking behaviors were excluded from the analysis based on their low prevalence and minimal relevance to depression among Korean women. Consistent findings from prior research and comprehensive literature reviews have demonstrated that these behaviors are infrequent and exert a negligible effect on depressive experiences in this population.
3. Household perception and attitude factors include five variables: satisfaction with the spouse’s contribution to housework, happiness with marital life, thoughts about husband, family decision-making, and family values.
  - Satisfaction with the spouse’s housework was measured on a 5-point Likert scale (1 = not satisfied at all; 5 = very satisfied), with higher scores indicating greater satisfaction.
  - Happiness with marital life was initially measured on a 10-point Likert scale but was converted to a scale of 100 for analysis, with higher scores indicating greater marital happiness.
  - Thoughts about husband was assessed using a 4-item scale: “I talk with my husband often”, “My husband and I share similar views”, “I am satisfied with our marital life (sexual relations)”, and “I trust my husband”. Each item was rated on a 4-point Likert scale (1 = not at all; 4 = very much so), with higher scores indicating more positive thoughts about one’s husband. The reliability coefficient (Cronbach’s α) for this scale was 0.825.
  - Family decision-making was assessed using an eight-item scale: my own employment, husband’s employment, my job change, husband’s job change, investment and asset management, household expense management, and family leisure activities. One point was assigned if the respondent was the primary decision-maker for each item; all other responses and missing responses (not applicable) were scored as 0. The total score ranged from 0 to 8, with higher scores indicating greater authority in family decision-making. The reliability coefficient (Cronbach’s α) for this scale was 0.631.
    Family values were assessed using a 14-item scale consisting of 5 forward-scored items (“Marriage is necessary”, “Marriage should be with someone from a similar background”, “It is better to marry early”, “It is good to have children early after marriage”, “Children are essential”) and 9 reverse-scored items (“Even with children, divorce is an option”, “Sexual relations without the premise of marriage are acceptable”, “Cohabitation without the premise of marriage is acceptable”, “Having and raising a child without marriage is possible”, “My personal achievements are more important than marriage”, “Marriage restricts my life”, “Sexual satisfaction is important in marital life”, “It is necessary to have opposite-sex friends besides one’s husband”, and “Divorce should happen if a husband cheats”). Each item was rated on a 4-point Likert scale (1 = strongly agree; 4 = strongly disagree), with higher scores indicating more modern family values. The reliability coefficient (Cronbach’s α) for this scale was 0.657.
4. Workplace perception and attitude factors include employment type, job satisfaction, and work–family balance.
  - Employment type was coded as 1 for regular positions and 0 for non-regular positions.
  - Job satisfaction was assessed using a 10-item scale that covered wage/income level, job security, job content, working environment, working hours, personal development opportunities, workplace communication and interpersonal relationships, welfare benefits, recognition for performance, and overall job satisfaction. Each item was rated on a 5-point Likert scale (1 = very dissatisfied; 5 = very satisfied), with higher scores indicating greater job satisfaction. The reliability coefficient (Cronbach’s α) for this scale was 0.901.
  - Work–family balance was assessed using 9 out of 11 items from the Korean Longitudinal Survey of Women and Families. Of the nine items, five pertained to the work-to-family domain (with items 4 and 5 reverse-coded): (1) Work gives me a sense of fulfillment and energy, (2) I think I can gain more recognition from my family by working, (3) Working makes family life more satisfying, (4) Long working hours interfere with family life, and (5) Irregular work hours disrupt family life. The remaining four items pertained to the family-to-work domain (with items 8 and 9 reverse-coded): (6) I work harder because I have a responsibility to support my family, (7) I work harder because my family appreciates what I do, (8) Having too much housework often makes my job difficult, and (9) I have considered quitting my job because of a family member’s illness. Each item was rated on a 4-point Likert scale (1 = strongly agree; 4 = strongly disagree), with higher scores indicating higher levels of conflict related to work–family balance. The reliability coefficient (Cronbach’s α) for this scale was 0.654.

### 2.4. Data Analysis

Data analysis was performed using SPSS 27.0 and R 4.0.0 software. Initially, frequency analysis and descriptive statistics were conducted to examine the characteristics of sociodemographic factors, health perception and behavior factors, household perception and attitude factors, and workplace perception and attitude factors, as related to the frequency of depressive experiences. This step involved calculating frequencies, percentages, means, and standard deviations.

To address the overdispersion observed in the depressive experience data (the dependent variable), the Akaike information criterion and Bayesian information criterion were used to evaluate the goodness-of-fit between the negative binomial model and the Poisson model. Given the high proportion of zero responses in the data, Vuong’s test was applied to compare the relative fit of the ZINB regression model against the standard negative binomial model. Additionally, a likelihood ratio test was conducted to further confirm the fit of the ZINB model.

Subsequently, a ZINB regression model was employed. The count model analysis was utilized to determine the influence of sociodemographic factors, health perception and behavior factors, household and workplace perception, and attitude factors on the frequency of depressive experiences among participants who reported experiencing depression. Additionally, the logit model analysis was used to identify factors associated with the likelihood of experiencing depression among those who reported no depressive experiences (i.e., zero frequency or no occurrence).

### 2.5. Ethical Considerations

This study was conducted in accordance with ethical guidelines after receiving raw data from the Korean Women’s Development Institute’s Korean Longitudinal Survey of Women and Families. The study protocol was reviewed and exempted by the Institutional Review Board (IRB) of the researcher’s affiliated university (IRB No. GIRB-D24-NX-0029).

## 3. Results

### 3.1. Distribution of Depressive Experience Scores and Model Fit Testing

**Figure 2:**
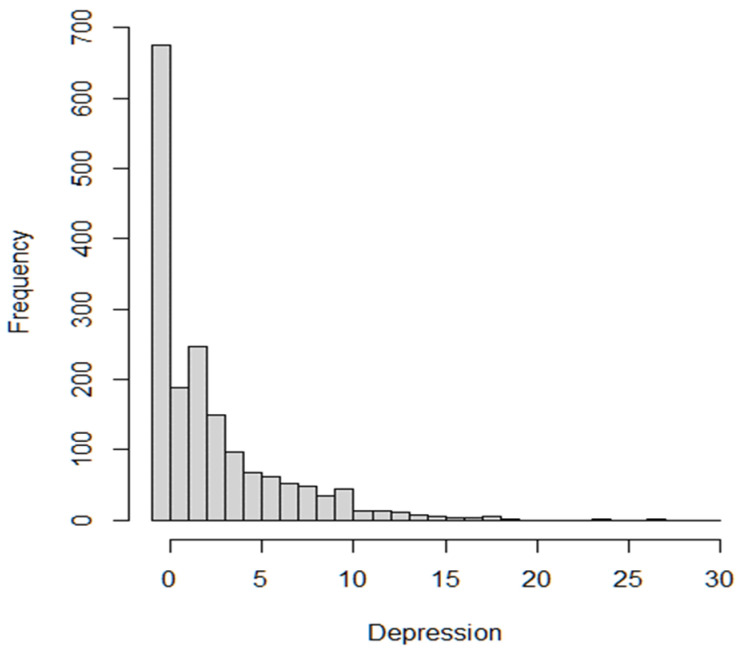
The frequency of depressive experiences among the study participants.

Of the 1735 participants in this study, 675 (38.9%) reported no depressive experiences. As Figure 2 illustrates, the overall distribution of scores showed a left-skewed, zero-inflated pattern. The mean depressive experience score was 2.71 ± 3.59, with a variance of 12.91. The likelihood ratio test confirmed the model’s significance (*p* < 0.001), and the explanatory power of the model was calculated at 67.7%.

### 3.2. Descriptive Statistics of Major Variable

Table 1 presents the frequency and descriptive analysis results of the participants’ sociodemographic factors, health perception and behavior factors, and household and workplace attitudes. The average age of the participants was 49.99 ± 7.38 years. The most frequent education level was college or higher (*n* = 858; 49.5%), followed by high school (*n* = 761; 43.9%), with middle school or lower accounting for only 6.7% (*n* = 116). The education level of the participants’ spouses followed a similar pattern: 987 (56.9%) had a college degree or higher, 646 (37.2%) had a high school education, and 102 (5.9%) had middle school education or lower. In terms of health perception and behavior factors, 81 participants (4.7%) rated their subjective health as poor, 505 (29.1%) as average, and 1149 (66.2%) as good. Regarding physical activity, 1158 participants (66.7%) reported no physical activity, while 577 (33.3%) reported engaging in physical activity.

Among the variables for household perception and attitudes, satisfaction with the spouse’s contribution to housework averaged 2.77 ± 0.90 on a 5-point scale, happiness with marital life 66.75 ± 14.48 on a 100-point scale, thoughts about husband 2.92 ± 0.46 on a 4-point scale, family decision-making authority 2.81 ± 1.62 on a 7-point scale, and family values 2.57 ± 0.31 on a 4-point scale. Regarding workplace perception and attitude factors, the average job satisfaction score was 3.49 ± 0.52 on a 5-point scale, and the average work–family balance score was 2.99 ± 0.36 on a 4-point scale.

### 3.3. Factors Influencing and Predicting Frequency of Depressive Experiences in Married Working Women

Table 2 presents the results of the ZINB regression model analysis of factors associated with the frequency of depressive experiences among married working women. The analysis is divided into two models, the count model and the logit model, each providing valuable insights into different aspects of depressive experiences.

In the count model analysis, subjective health status, a variable related to health perception and behavior factors, was significantly associated with the frequency of depressive experiences. Participants with average (*β* = −0.625, *p* < 0.001) and good (*β* = −0.864, *p* < 0.001) health reported significantly lower frequencies of depressive experiences compared to those with poor health. These results indicate that self-perceived health plays a significant role in the frequency of depressive experiences among married working women. By contrast, physical activity (*β* = 0.131, *p* = 0.049) was found to be positively correlated with a higher frequency of depressive experiences. In household perception and attitude factors, two variables stood out as significantly linked to a lower frequency of depressive experiences. More positive thoughts about one’s husband (*β* = −0.265, *p* < 0.001) and greater family decision-making authority (*β* = −0.079, *p* < 0.001) were both associated with reduced depressive experiences. These findings show that the more positive the perception of one’s spouse and the greater the authority in family decision-making, the more significant the role in alleviating depressive experiences among married working women. Moreover, in the workplace perception and attitude factors, work–family balance (*β* = −0.499, *p* < 0.001) emerged as a significant factor. Women who reported a better balance between work and family responsibilities experienced fewer depressive symptoms, highlighting the importance of supportive work environments that allow for better integration of personal and professional roles.

The logit model, which predicts the likelihood of depressive experiences among those reporting no depressive experiences (i.e., zero frequency), showed that participants whose spouses had a high school education (*β* = −0.843, *p* = 0.021) were more likely to experience depression compared to those whose spouses had middle school education or lower. Additionally, among health perception and behavior factors, physical activity (*β* = 0.373, *p* = 0.017) was found to significantly reduce the likelihood of experiencing depression, indicating that engaging in regular physical activity could serve as a protective factor against depression. In terms of household perception and attitude factors, satisfaction with the spouse’s contribution to housework (*β* = 0.253, *p* = 0.005) and marital happiness (*β* = 0.028, *p* < 0.001) were both significantly associated with a reduced likelihood of experiencing depression. This shows that positive marital relationships and the equitable distribution of domestic responsibilities may play a crucial role in preventing depressive experiences among married working women.

## 4. Discussion

This study aimed to identify factors influencing and predict the frequency of depressive experiences among married working women, with the goal of providing foundational data for developing strategies and programs to prevent and manage depression among this population. Variables were analyzed across four categories: sociodemographic factors, health perception and behavior factors, household perception and attitude factors, and workplace perception and attitude factors. The discussion in this section is based on the data analysis results.

The count model analysis revealed that married working women with a positive perception of their health experienced depression less frequently than those who rated their health as poor. This finding aligns with previous studies demonstrating that positive health perceptions have a beneficial effect on depression in married working women [15,27]. This suggests that positive subjective health perceptions can help mitigate depression through self-efficacy, positive thinking, health management behaviors, and increased social activity [28,29]. Therefore, to prevent depression in this population, it is crucial to develop diverse programs and implement policy efforts that help married working women perceive themselves as physically and mentally healthy.

Meanwhile, physical activity was identified as a factor that significantly increased the frequency of depressive experiences among married working women, which contradicts prior research indicating that physical activity has a positive effect on reducing depression [30,31]. However, some studies suggest that excessive physical activity may over-activate the hypothalamic–pituitary–adrenal axis, leading to physical stress and inflammatory responses, thereby worsening symptoms of depression and anxiety [32,33,34].

Additionally, White et al. [35] found that physical activity driven by autonomous motivation positively impacts mental health, while physical activity stemming from controlled motivation may have a negative impact. In this context, it is possible that married working women, who must juggle household and job responsibilities, may experience depression through physical activity in ways similar to those described in prior studies. Referring to the inconsistency in results, Huang et al. [36] argued that this variation may result from a lack of standardized and objective tools to measure physical activity in studies, as different studies employ varying methods and tools. In this study, physical activity was measured using a single item scale assessing the intensity of physical activity in the past week, potentially limiting the precision of the assessment. Therefore, caution is warranted when interpreting the results of this study regarding the relationship between physical activity and depression.

Our analysis also revealed that the more positively married working women perceive their husbands, the lower the frequency of depression. This finding is supported by Lee and Song [12] and Jeong et al. [37], who demonstrated that a wife’s more positive perception of her husband is directly associated with reduced depression. This can be attributed to the increased dependence on spouses in modern nuclear family households, where the spouse, as a life companion, has a greater impact on mental health than other family members do [37]. Therefore, helping married working women cultivate a cooperative home atmosphere would help reduce their stress and depression by fostering mutual respect and understanding, reducing conflict, and providing psychological stability through healthy communication with their spouse.

This study also found that married working women with greater household decision-making authority experience a lower frequency of depression. This finding aligns with prior research suggesting that a proactive role in the household positively influences mental health and helps prevent depression [38,39,40]. Similarly, some studies suggest that married women with limited or low decision-making authority are more likely to experience stress and depression owing to a lack of control over their living conditions [41,42]. However, other studies suggest that having more decision-making authority may also increase household burdens, negatively affecting mental health [43]. Therefore, additional replication studies with comparable population groups are needed to generalize these findings.

Lastly, this study found that married working women who manage work–family balance more effectively experience depression less frequently. This finding is consistent with previous research identifying work–family balance as a significant factor associated with depression [4]. This may be explained by Yucel and Borgmann’s [44] finding that married working women are more likely to experience negative emotions when they struggle to fulfill their dual roles in the workplace and at home, owing to work–family imbalance. For example, married working women may experience pressure, guilt, anxiety, and depression when they are unable to fulfill their role as a mother because of excessive work responsibilities [45,46] or when household duties prevent them from properly performing at work [44,47]. Therefore, there is a compelling need to establish practical support measures that enhance understanding and support within the workplace, as well as encourage cooperation and support from family members at home, empowering married working women to manage their multiple roles more effectively and prevent depression.

The logit model analysis of the ZINB regression revealed that married working women whose spouses had a high school education had a higher likelihood of experiencing depression. This finding aligns with prior studies indicating that lower education levels among spouses increase the risk of depression in women [48,49]. In South Korea, a college degree or higher is generally considered a high level of education, and individuals with only a high school diploma may face barriers to upward mobility, owing to lower employment rates, reduced opportunities for permanent employment, and wage disparities [50,51]. This labor market environment may lead spouses with a high school education to perceive themselves as less educated. A spouse’s low education level is directly associated with economic instability, limited emotional support, and reduced problem-solving capabilities, thereby increasing household stress and conflicts, which ultimately intensifies depression [48,49]. The results of this study can be interpreted within this context.

Physical activity was found to significantly reduce the likelihood of married working women experiencing depression, aligning with the findings of Farren et al. [52], which showed that physical activity effectively improves cardiorespiratory and overall fitness, thereby reducing depression levels. Additionally, physical activity was reported to reduce depression directly by lowering stress and promoting the secretion of key neurotransmitters like serotonin, dopamine, and norepinephrine [53].

However, in the count model analysis of this study, physical activity was identified as a factor that increased the frequency of depressive experiences. This discrepancy reflects the inherent difference between the count model, which analyzes factors affecting the frequency of depressive experiences, and the logit model, which examines predictors for not experiencing depression. It also underscores the inherent complexity in the association between physical activity and depression during the late pandemic period. The restrictive conditions imposed by the pandemic likely disrupted habitual routines and constrained opportunities for physical activity among married working women, potentially resulting in deviations from outcomes expected under normal circumstances. Additionally, the unavailability of reliable measurement tools for physical activity may have had a considerable impact on the observed relationship between physical activity and depressive symptoms. A further limitation of the present study lies in its reliance on secondary data, which inherently precluded the inclusion of critical psychosocial constructs, such as self-efficacy and social support, and key characteristics of physical activity that are well established predictors of mental health outcomes. Recognizing the potential influence of these unmeasured variables, future investigations should adopt a more holistic methodological approach, incorporating robust assessments of physical activity and salient psychosocial variables to provide a more nuanced understanding of the intricate relationship between physical activity and depression.

In the logit model analysis, higher satisfaction with the spouse’s contribution to housework was found to lower the likelihood of depression among married working women, supporting the findings of previous research [54]. This can be interpreted as indicating that reducing the gap between perception and practice in gender equality—moving away from traditional gender role divisions—may mitigate the negative effects of these disparities on depression [55,56]. Therefore, actively encouraging husbands’ participation in housework and raising societal awareness about gender equality are essential for reducing depression among married working women.

Regarding married working women’s perception of marital life, it was found that greater happiness in marital life was associated with a lower likelihood of experiencing depression. This finding is supported by several studies that have identified marital happiness as a factor associated with depression in this population [12,57]. Previous studies have reported that a satisfying relationship with a spouse, which leads to higher marital happiness, strengthens emotional support and serves as a protective factor against depression, even in stressful situations [58,59,60]. Therefore, strategies to enhance marital happiness are crucial for preventing depression among married working women. This underscores the need for systematic support, such as counseling and education, to improve satisfaction and happiness in married life, ultimately promoting the overall well-being of families.

This study has several limitations. The cross-sectional design, while useful for identifying associations, limits the ability to establish causal relationships between depression and related factors. Moreover, the observed relationships among the variables may exhibit bidirectionality, wherein depression is not only correlated with certain factors, but may also act as both an antecedent and a consequence within these associations. Future research should adopt a longitudinal approach to better explore these causal links and account for the potential of reverse causation. The reliance on secondary data also restricted the precision of variable selection, as certain important factors were not captured, including self-efficacy, social support, physical activity characteristics, mental health history, sleep quality, and hormonal changes. This highlights the potential value of primary data collection to improve the accuracy and comprehensiveness of future analysis. Additionally, the absence of a comparison group, such as non-working married women, limits the generalizability of the findings. Including a comparison group and expanding the range of demographic variables, such as occupational background and family structure, could provide deeper insights into the factors contributing to depression.

Despite these limitations, the significance of this study is underscored by its provision of practical foundational data essential for the development of policies and programs aimed at mitigating and preventing depression problems among married working women. This is achieved through a multifaceted analysis using the ZINB regression analysis method to examine the influencing and predictive factors associated with depressive experiences of married working women.

## 5. Conclusions

This study utilized the ZINB regression model, which effectively addressed the overdispersion and asymmetry of depression data that are challenging to manage in traditional research. This model provided highly accurate estimates from the skewed depression data, improving the reliability of the study’s results and deepening the understanding of depression among married working women. Additionally, the various associated factors derived in this study can serve as critical data for developing strategies and policies to alleviate depressive experiences. The findings of this study indicate that enhancing health perceptions, promoting physical activity, and improving work–family balance are pivotal strategies for advancing the mental health of married working women. Moreover, the results demonstrate that being able to have a more positive perception of one’s spouse and increasing decision-making authority within the family can play a significant role in mitigating depressive symptoms. A particularly noteworthy discovery was the identification of satisfaction with household labor division and marital happiness as key predictive factors for participants who reported no depressive experiences. These insights highlight the critical importance of targeting these factors as part of tailored, evidence-based interventions for married working women.

Consequently, this study provides essential data for the development of targeted interventions aimed at alleviating depressive experiences among married working women. Such interventions, which a focus on promoting health, increasing physical activity, and enhancing work–family balance, have the potential to significantly improve both the quality of life and productivity of this population. Additionally, the integration of mental health support into marriage counseling and family relationship programs would facilitate more comprehensive mental health care for married working women. By addressing these factors, the proposed strategies offer sustainable and effective solutions to the mental health challenges faced by this demographic, with the potential for long-term positive impacts at both the individual and societal levels.

## Figures and Tables

**Table 1:** Descriptive statistics of major variables (*N* = 1735).

Factors	Variable		Category	n	%	M ± SD
Sociodemographic Factors	Age (years)					49.99 ± 7.38
	Education		≤Middle school	116	6.7	
			High school	761	43.9	
			College graduate≥	858	49.5	
	Spouse’s Education		≤Middle school	102	5.9	
			High school	646	37.2	
			College graduate≥	987	56.9	
	Monthly Income (10,000 KRW)					221.80 ± 122.48
	Number of Preschool Children					0.09 ± 0.34
	Number of School-Aged Children					0.63 ± 0.88
Health Perception and Behavior Factors	Subjective Health Status		Poor	81	4.7	
			Average	505	29.1	
			Good	1149	66.2	
	Physical Activity (1 week)		No	1158	66.7	
			Yes	577	33.3	
	BMI (kg/m^2^)					22.54 ± 2.59
	Body Shape Perception		Thin	138	8	
			Average	1135	65.4	
			Obese	462	26.6	
Household Perception and Attitudes	Satisfaction with Spouse’s Housework					2.77 ± 0.90
	Happiness with Marital Life					66.75 ± 14.48
	Thoughts About Husband					2.92 ± 0.46
	Family Decision-Making					2.81 ± 1.62
	Family Values					2.57 ± 0.31
Workplace Perception and Attitudes	Employment Type	Regular		899	51.8	
		Non-Regular		836	48.2	
	Job Satisfaction					3.49 ± 0.52
	Work–Family Balance					2.99 ± 0.36

**Table 2:** Zero-inflated negative binomial regression for frequency of depressive experiences (*N* = 1735).

Variables		Count Model					Logit Model				
		B	SE	Z	*p*	95% CI	B	SE	Z	*p*	95% CI
Sociodemographic Factors	Age (years)	−0.006	0.006	−0.932	0.351	−0.018~0.006	−0.015	0.015	−0.968	0.333	−0.045~0.015
	Education (Ref: ≤middle school)										
	High School	−0.095	0.136	−0.697	0.486	−0.362~0.172	0.275	0.391	0.705	0.481	−0.490~1.041
	College Graduate≥	−0.102	0.151	−0.673	0.501	−0.399~0.195	0.147	0.430	0.342	0.732	−0.696~0.990
	Spouse’s Education (Ref: ≤ middle school)										
	High school	−0.144	0.146	−0.989	0.323	−0.430~0.142	−0.843	0.365	−2.309	0.021	−1.560~−0.127
	College graduate≥	−0.055	0.158	−0.346	0.729	−0.364~0.255	−0.734	0.390	−1.880	0.060	−1.499~0.031
	Monthly Income (10,000 KRW)	<0.001	<0.001	−0.195	0.845	−0.001~0.001	<0.001	0.001	−0.262	0.793	−0.002~0.001
	Number of Preschool Children	−0.111	0.115	−0.968	0.333	−0.336~0.114	0.038	0.244	0.154	0.878	−0.441~0.516
	Number of School-Aged Children	0.002	0.040	0.057	0.955	−0.076~0.080	−0.190	0.103	−1.845	0.065	−0.392~0.012
Health Perception and Behavior Factors	Subjective Health (Ref: Poor)										
	Average	−0.625	0.119	−5.234	<0.001	−0.858~−0.391	0.357	0.369	0.967	0.333	−0.366~1.079
	Good	−0.864	0.117	−7.395	<0.001	1.093~−0.635	0.660	0.358	1.844	0.065	−0.042~1.361
	Physical Activity (Ref: No)										
	Yes	0.131	0.067	1.967	0.049	<0.001~0.262	0.373	0.156	2.384	0.017	0.066~0.679
	BMI (kg/m^2^)	0.017	0.015	1.196	0.232	−0.011~0.046	0.007	0.038	0.194	0.846	−0.067~0.082
	Body Shape Perception (Ref: Thin)										
	Average	−0.156	0.115	−1.361	0.173	−0.381~0.069	−0.024	0.278	−0.085	0.933	−0.569~0.522
	Obese	−0.149	0.137	−1.091	0.275	−0.417~0.119	−0.572	0.351	−1.630	0.103	−1.260~0.116
Household Perception and Attitudes	Satisfaction with Spouse’s Housework	0.056	0.034	1.663	0.096	−0.010~0.121	0.253	0.091	2.779	0.005	0.075~0.431
	Happiness with Marital Life	0.002	0.002	0.838	0.402	−0.003~0.007	0.028	0.007	4.071	<0.001	0.015~0.042
	Thoughts about Husband	−0.265	0.076	−3.492	<0.001	−0.414~−0.116	0.318	0.196	1.621	0.105	−0.067~0.702
	Family Decision-Making	−0.079	0.019	−4.235	<0.001	−0.115~−0.042	0.090	0.048	1.876	0.061	−0.004~0.185
	Family Values	−0.087	0.095	−0.910	0.363	−0.274~0.100	−0.221	0.248	−0.890	0.373	−0.707~0.265
Workplace Perception and Attitudes	Employment Type (Ref: Regular)										
	Non-Regular	0.020	0.067	0.302	0.763	−0.112~0.153	0.020	0.171	0.115	0.908	−0.316~0.355
	Job Satisfaction	−0.110	0.065	−1.694	0.090	−0.237~0.017	0.267	0.164	1.631	0.103	−0.054~0.587
	Work–Family Balance	−0.499	0.081	−6.142	<0.001	−0.658~−0.340	−0.033	0.209	−0.156	0.876	−0.443~0.377

## Data Availability

The dataset used and/or analyzed during this study can be provided by the corresponding author on reasonable request.
